# Physical exercise, sport, psychiatry, and mental health

**DOI:** 10.1002/pcn5.70300

**Published:** 2026-02-17

**Authors:** Driss Moussaoui, Ira D. Glick

**Affiliations:** ^1^ Department of Psychiatry Ibn Rushd University Psychiatric Center Casablanca Morocco; ^2^ World Association of Social Psychiatry New Delhi India; ^3^ World Federation for Psychotherapy New York USA; ^4^ Stanford University School of Medicine Stanford California USA; ^5^ National Institute of Mental Health Bethesda Maryland USA

**Keywords:** mental disorders, physical exercise, prevention, sport, treatment

## Abstract

In Psychiatry and Mental Health, physical exercise and sport have been neglected as essential tools in preventing the occurrence of mental disorders, in improving health and mental health, and in treating mental patients. This paper advocates for a better knowledge of this field by mental health workers in the best interest of care of mental patients.

A revolution is on its way, both in the field of Psychiatry and Mental Health, that is, symptoms, problems, and disorders.^1^, ^2^ Physical activity and sports are useful for improving health in general, but especially in Psychiatry and Mental Health, from both the preventive and therapeutic points of view.

This behavior was known more than 2400 years ago: Aristotle wrote that he thought better while walking, as compared to being seated or sedentary. The Latin sentence “Mens sana in corporeo sano” (A healthy mind in a healthy body) is well known since the Roman Empire. However, it was not seen as relevant in Psychiatry, and for centuries, it was adapted exactly the opposite way. One of the authors (D.M.) had a teacher in France who told the following story: “In 1945, he became chief of a ward of chronic psychotic patients. He saw all the mental patients lying on the bed. When he asked why are they just laying there—the answer of the nurses was: ‘Because they are ill’. When he insisted to encourage them to go out in the garden, the nurses threatened a strike, before things settled in a positive way, i.e. getting them moving.”

Physical exercise/playing sports has a beneficial effect on the body, that is, thought to prevent and helping treat obesity, diabetes, high blood pressure, and cancer, among other illnesses.^3^ It reinforces the heart and respiratory system, muscles, joints, and the immune system, as well as improving the sense of balance, especially with cycling. Let us remind that about a third of elderly people die directly or indirectly from a fall. On the psychological/psychiatric side, physical activity and sports also have a significant anti‐stress, anti‐anxiety disorders, and anti‐depressive effect, as well as an anti‐Alzheimer Disease effect—all preventive and potentially therapeutic. There is no known medication that has as much impact on health and mental health at the same time.

The physiology of such effects on the brain is thought to be mediated by the secretion of myokines by the muscles, among other biological changes in the body. The myokines cross the blood–brain barrier and produce the brain‐derived neurotrophic factor (BDNF). The consequence of that, exactly like with antidepressant medications and other antidepressant treatments, is the production of new neurons and synapses (neoneurogenesis). This remarkable effect may start with as little as 30 min session.

Practically speaking, we suggest that mental health workers themselves should learn about the preventive effect of physical exercise/sport on body and mind illnesses and disorders, and also learn how to use them therapeutically. Further, we encourage them to practice one or more sports themselves, to have the experience of better cognition and mood. We suggest they will be good advocates of this good cause when they see such effects in their own lives.

Importantly, we suggest to systematically explore physical activity with every patient, and to motivate them, going into detail (help with connected watches, duration, and effort during sessions of exercise). It is fascinating to see the changes that happen in patients who have improved partially with medications when they exercise in a regular way. Rarely does sport become an addiction. If it happens, it has to be treated.

Age is not a limiting factor. A number of people start doing sports at the age of 70. This is possible with the agreement of a cardiologist, but there is an obligation to “start slow and be progressive.” The two authors (D.M., age 76, and I.D.G., age 90) continue to have sports activity on a regular basis (running, cycling, swimming, and basketball) and experience clearly the cognitive and body impact of sports. One of us (I.D.G.) is the oldest player in the United States still playing full‐court basketball regularly.

Last but not least, it is important to have favorable policies relating to sport nationally. For example, recently, the Spanish parliament passed a law to teach all students to use a bicycle in primary schools. They insisted that from now on, no urban development could be started without cycling corridors in cities or in rural areas. This has two benefits: healthy activity for the individuals, whether for leisure or for commuting. Less use of cars leads to less pollution of the air, which is also good for the health of the community.

In summary, health workers, including those in the mental health field, need to implement this increasing knowledge from research, aimed towards (a) further training and education of the community, (b) involvement of lawmakers, (c) promoting good health, (d) decreasing mental disorders, and (e) treating with a sport when indicated.^4^

## AUTHOR CONTRIBUTIONS

Both authors contributed.

## CONFLICT OF INTEREST STATEMENT

The authors declare no conflicts of interest.

## ETHICS APPROVAL STATEMENT

N/A.

## PATIENT CONSENT STATEMENT

N/A.

## CLINICAL TRIAL REGISTRATION

N/A.

## Data Availability

N/A.
